# Allergic Sensitization to Common Ragweed and Mugwort Pollen Allergens: A 6‐Year Single‐Center Retrospective Analysis

**DOI:** 10.1155/jimr/9654422

**Published:** 2026-06-22

**Authors:** Zhipeng Zhao, Runqing Li, Rui Li, Pan Xiong, Xiaochen Li, Hongjing Liu, Yanhua Dai

**Affiliations:** ^1^ Department of Laboratory Medicine, Beijing Tsinghua Changgung Hospital, School of Clinical Medicine, Tsinghua Medicine, Tsinghua University, Beijing, China, tsinghua.edu.cn

**Keywords:** *Ambrosia artemisiifolia*, *Artemisia vulgaris*, common ragweed (*Ambrosia*), cross-reaction, ecologically invasive plant, mugwort (*Artemisia*), pollen allergy, sIgE

## Abstract

**Objective:**

Based on a 6‐year longitudinal monitoring of specific immunoglobulin E (sIgE) responses to common ragweed (*Ambrosia*, *Ambrosia artemisiifolia*) and mugwort (*Artemisia*, *Artemisia vulgaris*) pollen allergens, this study systematically analyzed the sensitization trends of these two prevalent aeroallergen sources. The findings aim to provide evidence‐based references for effective screening, early prevention, and targeted intervention in high‐risk populations.

**Methods:**

This study retrospectively analyzed the test results of 2798 patients who underwent both common ragweed pollen‐sIgE and mugwort pollen‐sIgE testing at Beijing Tsinghua Changgung Hospital from January 2020 to December 2025. It explored the sensitization characteristics of these two allergens across different time periods, age groups, and genders and performed a comparative analysis of their sensitization profiles.

**Results:**

The case volume peaked in 2025, followed by 2022. The positive rates of common ragweed and mugwort pollen‐sIgE exhibited a decreasing trend from 2020 to 2024, yet rebounded in 2025. The highest positive rate was observed in the 6–15 years age group, with the overall positive rate showing a downward trend as age increased. The number of male patients was significantly higher than that of female patients (*χ* ^2^ = 15.22, *p* < 0.01). Moreover, the positive rates of common ragweed and mugwort pollen‐sIgE were significantly higher in males than in females (common ragweed: *χ* ^2^ = 9.17, *p* < 0.01; mugwort: *χ* ^2^ = 15.22, *p* < 0.01). Among all positive cases (*n* = 673), 91.23% were sensitized to mugwort pollen allergens, 64.19% to common ragweed pollen allergens, and 55.42% to both allergens. For patients with dual positivity to common ragweed and mugwort pollen allergens (*n* = 373), the detection levels of mugwort‐specific IgE were significantly higher than those of common ragweed‐sIgE (*p* < 0.01).

**Conclusions:**

In 2025, the positive rates of common ragweed pollen‐sIgE and mugwort pollen‐sIgE in the Beijing area exhibited a rebound trend. Positive rates of common ragweed and mugwort pollen‐sIgE decreased with increasing age, whereas they were significantly higher in males than in females. Mugwort pollen allergens demonstrated stronger allergenicity than common ragweed pollen allergens; nevertheless, the two allergens showed highly consistent sensitization trends. These findings also corroborate the cross‐reactivity between common ragweed and mugwort pollen allergens documented in the literature. However, further investigations are warranted to characterize the specific cross‐reactive components and to develop novel diagnostic reagents.

## 1. Introduction

Allergic diseases are among the most prevalent disorders worldwide, affecting populations across all age groups. The World Health Organization (WHO) has identified them as a priority area for disease prevention and control in the 21st century. Although there is no single definitive global dataset on the incidence of allergic diseases, it is widely recognized that more than 20% of the global population has suffered from allergic conditions at some point in their lives. The global prevalence of allergic rhinitis (AR) is estimated to range between 5% and 50%, showing a consistent upward trend. For instance, AR affects 20% of the population of the United Kingdom [1]. Beyond respiratory manifestations, allergies can exert systemic effects on the human body. Repeated daily measurements using wearable remote monitoring devices have confirmed that the daily allergic burden of AR in adults affects not only the respiratory system but also overall systemic health [2]. Globally, the prevalence of allergies presents a distinct socioeconomic gradient [3], with marked heterogeneity in allergen spectra and epidemiological characteristics across different regions [4]. Consequently, conducting region‐specific studies on pollen allergen‐related allergies is imperative to provide evidence‐based support for early prevention and clinical intervention. To elucidate the epidemiological trends of common ragweed and mugwort pollen allergen‐related allergies in Beijing, we conducted a retrospective analysis of 2798 clinical samples collected at the Beijing Tsinghua Changgung Hospital between 2020 and 2025. Serum‐specific immunoglobulin E (sIgE) levels against common ragweed and mugwort pollen were assessed. This study aims to characterize the recent sensitization patterns of these major allergens, thereby laying a theoretical foundation for the precise prevention and management of regional allergic diseases.

## 2. Materials and Methods

### 2.1. Subjects

This retrospective study analyzed sIgE testing data for common ragweed and mugwort pollen from patients evaluated at Beijing Tsinghua Changgung Hospital, Tsinghua University, spanning the period from 2020 to 2025. Only subjects that underwent simultaneous testing for both pollen‐sIgEs were included. For patients with multiple test records, the following criteria were applied: if the test results were consistent, only the earliest record was retained. If the results were inconsistent (i.e., discordant), two specific records were preserved: the earliest negative result and the earliest positive result. The mean age of the study population (individuals who underwent sIgE testing for common ragweed and mugwort pollen allergens) was 34.21 years (range: 1–94 years). Among these subjects, there were 1497 males (53.60%) and 1301 females (46.40%), as presented in Table 1.

**Table 1:** Baseline characteristics of the study population.

Gender	Number	Ratio (%)	*χ* ^2^	*p*
Male	1497	53.60	13.72	<0.01
Female	1301	46.40		

The research protocol was approved by the Ethics Committee of Beijing Tsinghua Changgung Hospital (Ethics Approval Number: 24761‐4‐01).

### 2.2. Laboratory Procedures

Four milliliters (4 mL) of peripheral venous blood was collected from each subject and centrifuged at 4000 revolutions per minute (rpm) for 5 min. The separated serum was stored at 2–8°C in a refrigerator and assayed within 3 days. Serum levels of sIgE were measured using the Phadia 250 automated fluorescence immunoassay system (Phadia AB, Uppsala, Sweden) equipped with ImmunoCAP technology.

### 2.3. Statistical Analysis

Serum‐sIgE concentrations were quantitatively reported in kilo units per liter (kU/L), with a reference cutoff value of <0.35 kU/L. Samples with sIgE levels ≥ 0.35 kU/L were defined as sensitization‐positive, whereas those below this threshold were classified as negative. Annual trend analysis assessed variations in case volume and sensitization positivity rates for common ragweed and mugwort pollen‐sIgE across the study years. The positive rates were subjected to a chi‐square test. Gender difference analysis: conducted comparative analyses of sensitization positivity rates between male and female subjects were conducted. Age‐stratified analysis: evaluated case volume and positivity rates across 5‐year age subgroups and analyzed sIgE quantitative levels among different age groups. Venn diagram analysis: visualized the distribution of common ragweed pollen allergen monosensitization cases, mugwort pollen allergen monosensitization cases, and dual‐sensitization cases (positive for both pollen allergens). Quantitative comparative analysis: Investigated differences in sIgE quantitative levels between common ragweed and mugwort pollen in dual‐sensitization cases.

Categorical variables (e.g., sensitization positivity rates) were analyzed using the chi‐square test, which was performed with SPSS software (Version 26.0; IBM Corporation, Armonk, New York, USA). The Mann–Whitney *U* test (for comparisons of nonnormally distributed continuous variables, e.g., serum‐sIgE levels) was conducted using GraphPad Prism software (Version 10; GraphPad Software, Boston, Massachusetts, USA). A two‐tailed *p*‐value < 0.05 was considered statistically significant.

## 3. Results

### 3.1. Temporal Distribution Patterns of Allergic Sensitization to Common Ragweed and Mugwort Pollen Allergens

**Figure 1:**
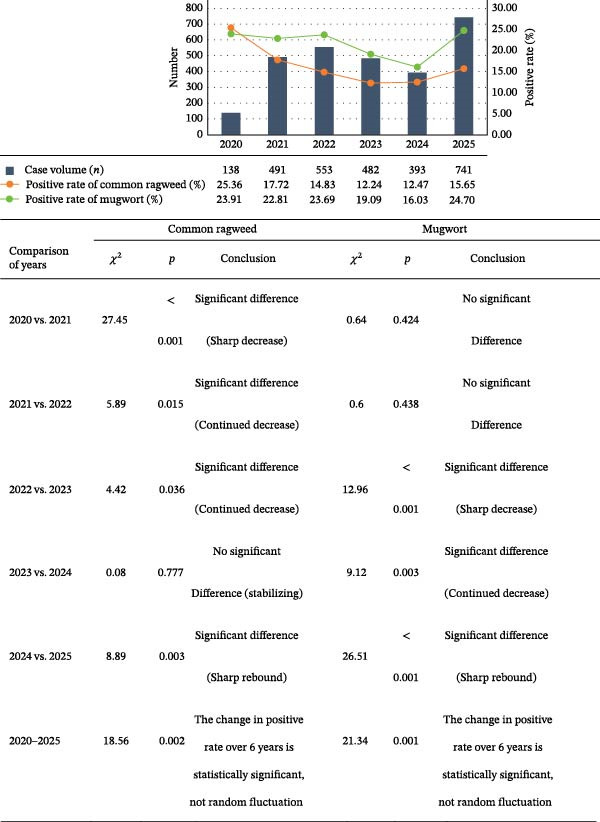
The annual case volume of testing and positive rates of common ragweed and mugwort pollen allergens.

The annual case volume from 2020 to 2025 was 138,491,553,482,393, and 741, respectively. Over the same period, the annual sensitization‐positive rates for common ragweed pollen allergens were 23.91%, 17.72%, 14.83%, 12.24%, 12.47%, and 15.65%, while those for mugwort pollen allergens were 25.36%, 22.81%, 23.69%, 19.09%, 16.20%, and 24.70%. The case volume peaked in 2025, followed by 2022. From 2020 to 2024, the sensitization‐positive rates of both allergens exhibited a consistent downward trend year on year, yet rebounds were observed in 2025 compared to 2024 (common ragweed: *p* = 0.003; mugwort: *p* < 0.001). Temporal variations in sensitization‐positive rates to common ragweed and mugwort pollen allergens were statistically significant over the 6‐year period from 2020 to 2025, excluding random fluctuations (common ragweed: *p* = 0.002; mugwort: *p* = 0.001). (Figure 1).

### 3.2. Gender‐Based Differences in Sensitization to Ragweed and Mugwort Pollen Allergens

Among the study participants, males accounted for a significantly higher proportion (*n* = 1497, 53.60%) than females (*n* = 1301, 46.40%), with this difference reaching statistical significance (*χ* ^2^ = 13.72, *p* < 0.01), as shown in Table 1.

The sensitization‐positive rate to common ragweed pollen allergens was significantly higher in males (17.37%) than in females (13.22%) (*χ* ^2^ = 9.17, *p* < 0.01). A similar gender disparity was observed for mugwort pollen allergens, where males also exhibited a significantly higher sensitization‐positive rate (23.78%) compared with females (19.83%) (*χ* ^2^ = 6.34, *p* < 0.05), as presented in Table 2.

**Table 2:** Gender differences in positive rates to common ragweed and mugwort pollen allergens.

Allergen	Gender (*n*)	Positive	Negative	*χ* ^2^	*p*
Common ragweed	Male (*n* = 1497)	260 (17.37%)	1237 (82.63%)	9.17	<0.01
	Female (*n* = 1301)	172(13.22%)	1129(86.78%)		
Mugwort	Male (*n* = 1497)	356(23.78%)	1141(76.22%)	6.34	<0.05
	Female (*n* = 1301)	258(19.83%)	1043(80.17%)		

For common ragweed pollen‐sIgE, the median level was identical at 0.03 kU/L in both males and females, whereas the corresponding mean levels were 0.67 kU/L and 0.57 kU/L, respectively. The Mann–Whitney *U* test showed that this intergender difference was statistically significant (*U* = 878102.00, *Z* = −4.521, *p* < 0.001). For mugwort pollen‐sIgE, the median level was 0.04 kU/L in males versus 0.03 kU/L in females, with the corresponding mean levels being 2.62 kU/L and 2.57 kU/L, respectively; the gender‐based difference was also statistically significant (*U* = 868748.000, *Z* = −4.952, *p* < 0.01). As detailed in Table 3.

**Table 3:** Gender differences in sIgE values to common ragweed and mugwort pollen allergens.

Statistical parameters	Common ragweed pollen‐sIgE (kU/L)		Mugwort pollen‐sIgE (kU/L)	
	Male (*n* = 1497)	Female (*n* = 1301)	Male (*n* = 1497)	Female (*n* = 1301)
Median	0.03	0.03	0.04	0.03
Mean	0.67	0.57	2.62	2.57
Range	0.00–34.70	0.00–74.80	0.00 −100.00	0.00 −100.00
*U*	878102.00		868748.00	
*Z*	−4.521		−4.952	
*p*	<0.01		<0.01	

### 3.3. Age‐Associated Differences in Sensitization to Ragweed and Mugwort Pollen Allergens

**Figure 2:**
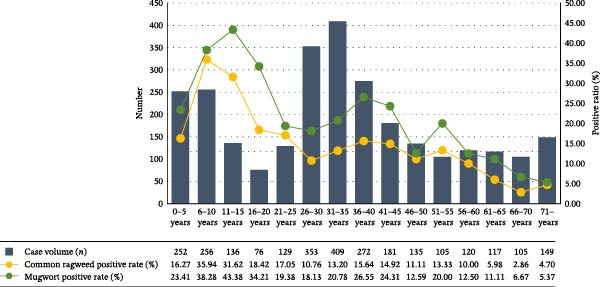
Age‐related changes in positive rates of common ragweed and mugwort pollen allergens. Enrolled cases were grouped in 5‐year increments, and those aged ≥70 years were pooled into one category.

The highest case volume was observed in the 26–40‐year‐old age group, followed by the 1–10‐year‐old pediatric subgroup. However, the sensitization rates for both common ragweed and mugwort pollen allergens peaked in the 6–15‐year‐old age group, with a secondary peak noted in the 36–45‐year‐old age group. Overall, the sensitization rates to the two allergens showed a significant age‐dependent downward trend, with the temporal patterns of common ragweed and mugwort pollen allergens being highly parallel. As shown in Figure 2.

### 3.4. Comparison of Sensitization Characteristics Between Common Ragweed and Mugwort Pollen Allergens

Among the 2798 enrolled cases, the overall sensitization‐positive rate to mugwort pollen allergens (21.90%) was significantly higher than that to common ragweed pollen allergens (15.10%). As shown in Figure 2, the sensitization‐positive rate to mugwort pollen allergens remained consistently higher than that to common ragweed pollen allergens across all age subgroups.

**Figure 3:**
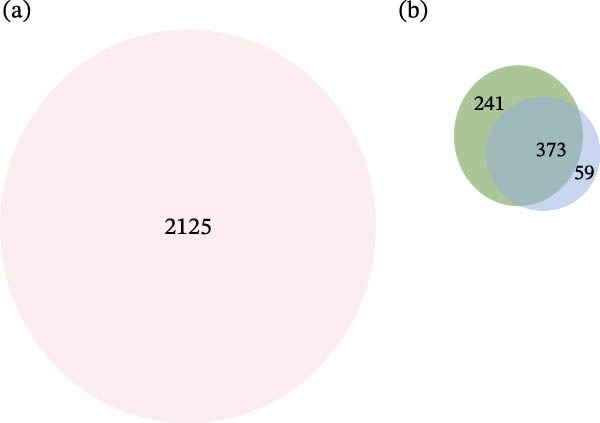
Distribution of sIgE levels for common ragweed and mugwort pollen allergens. Color coding: (a) pink, common ragweed (–)/mugwort (–) (2125 cases, 75.95%); (b) purple, common ragweed (+)/mugwort (–) (59 cases, 8.77%); blue, common ragweed (+)/mugwort (+) (373 cases, 55.42%); and green, common ragweed (–)/mugwort (+) (614 cases, 35.81%).

As shown in Figure 3, among all cases, 75.95% (2125/2798) were negative for both common ragweed and mugwort pollen allergens (pink area), whereas 673 subjects were positive for common ragweed and/or mugwort pollen allergens, corresponding to an overall cosensitization or single sensitization rate of 24.05%. Of these positive cases, 55.42% (373/673) were common ragweed pollen allergens (+)/mugwort pollen allergens (+) (blue area), 8.77% (59/673) were common ragweed pollen allergens (+)/mugwort pollen allergens (−) (purple area), and 35.81% (241/673) were common ragweed pollen allergens (−)/mugwort pollen allergens (+) (green area).

**Figure 4:**
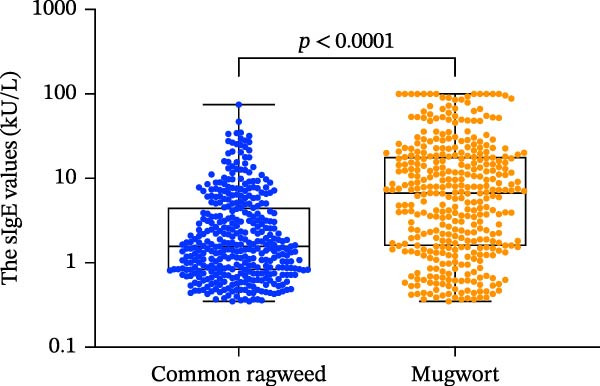
Distribution of sIgE levels against common ragweed and mugwort pollen allergens. Among 373 dual‐positive (common ragweed/mugwort) cases. Mugwort pollen allergen‐sIgE levels were significantly higher compared with those of common ragweed (mean difference = 9.762, SD = 19.62; 95% CI: 7.403–12.12; *p* < 0.01).

**Figure 5:**
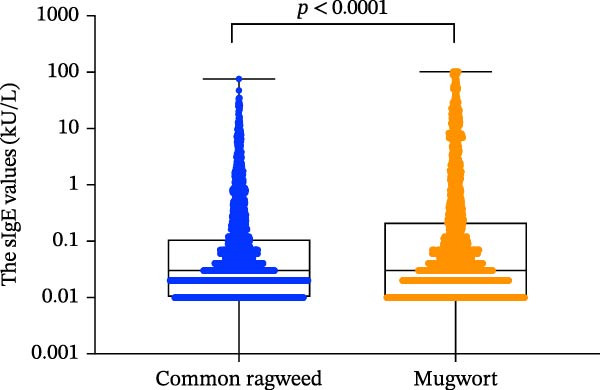
Distribution of sIgE levels against common ragweed and mugwort pollen allergens in 2798 cases. The levels of mugwort pollen allergen‐sIgE were significantly higher than those of common ragweed (mean difference = 1.505, SD = 7.68; 95% CI: 1.172–1.837; *p* < 0.01).

In the 373 common ragweed pollen allergens (+)/mugwort pollen allergens (+) cases, the mugwort pollen‐sIgE levels were significantly higher than the common ragweed pollen‐sIgE levels (mean difference = 9.762 kU/L, SD = 19.62; 95% CI: 7.403–12.12; *p* < 0.01), as shown in Figure 4. Moreover, among all 2798 enrolled subjects, the serum mugwort pollen‐sIgE levels were also significantly higher than the serum common ragweed pollen‐sIgE levels (mean difference = 1.505 kU/L, SD = 7.68; 95% CI: 1.172–1.837; *p* < 0.01), as shown in Figure 5.

## 4. Discussion

Allergy caused by pollen allergens is highly prevalent worldwide with remarkable regional disparities; the reported prevalence rates are 17.6% in China, 18.0% in the United States, and 40.0% in Europe, respectively [5–7]. Its occurrence is modulated by multiple factors, including flowering phenolog and, environmental conditions, as well as demographic and immunological characteristics such as gender, age, and immune status [8–12]. A clinical study conducted at the First People’s Hospital of Yinchuan further verified that the sensitization‐positive rates of common ragweed pollen allergens and mugwort pollen allergens exhibit significant variations across different age groups, genders, and ethnicities [6]. Common ragweed and mugwort are major sources of allergenic weed pollen. The sensitization‐positive rates to these two pollen allergens vary across different countries and regions, highlighting the necessity of investigating their regional‐specific sensitization profiles. Given the significant interregional and international variations in the sensitization characteristics of common ragweed and mugwort pollen allergens, this single‐center study was conducted in Beijing to explore temporal trends in their positive rates, age‐related differences, and gender‐based variations. Additionally, it examined potential cross‐reactivity between allergens from these two pollens with the aim of providing a more robust evidence base for the interpretation of allergen test results.

Allergic diseases have become a major public health concern, affecting over 25% of the population in industrialized countries. Notably, their prevalence is also increasingly rising in developing regions across the globe [13, 14]. The rising prevalence of allergic diseases presents a substantial public health challenge. Common ragweed and mugwort, both Asteraceae species, show distinct distribution patterns in China: the former, a North American invasive plant, is now global [15], while the latter has a long cultivation history and wide domestic distribution. Their pollens are major respiratory allergens, threatening public health in endemic regions. Based on skin prick test (SPT) data originating from Nepal, 11.8% of patients with RA in this region exhibited sensitization to common ragweed pollen allergens, and 10% were sensitized to mugwort pollen allergens [16]. In contrast, among patients with pollen allergen allergeis in Guangdong Province, southern China, the sensitization‐positive rates to common ragweed and mugwort pollen allergens were 40.6% and 37.0%, respectively [17]. Beijing Shijitan Hospital, which is located in the northern part of China, conducted a retrospective analysis of SPT data from allergic patients between January 2017 and December 2019, which revealed a 45.5% sensitization‐positive rate to common ragweed pollen allergens [18]. Additionally, single‐center data from our hospital (located in the city of Beijing, north of China) covering the period 2020–2025 demonstrated that the sensitization‐positive rates to common ragweed and mugwort pollen allergens were 15.54% and 21.90%, respectively. Annual variations were noted in the positive rates of common ragweed and mugwort pollen allergens. Our data demonstrated a consistent year‐on‐year decrease in the positive rates of these two allergens from 2020 to 2024; nevertheless, a notable rebound in their positive rates was recorded in 2025. Elevated pollen concentrations can induce new sensitization in previously nonallergic individuals, thereby increasing the number of newly diagnosed allergic patients [19, 20]. Meteorological reports sourced from authoritative institutions such as the National Meteorological Center, China Weather Network, and official media reports like Beijing Daily indicated that the pollen concentration in Beijing reached Grade 5 (≥800 grains/m^3^) for 15 consecutive days from August 26 to September 9, 2025. This level was higher than that recorded in 2023 and 2024, accompanied by a longer duration and wider spatial coverage of high pollen concentrations. Common ragweed and mugwort pollens are important components of autumn‐season airborne pollen in Beijing. Previous studies indicated that mugwort pollen is the most abundant autumn‐season pollen type in Beijing, accounting for 10.52% of total airborne pollen [21]. Also, ragweed pollen is highly sensitized in the Beijing area [22]. Long‐term monitoring revealed that peak mugwort pollen concentrations in urban Beijing ranged from 111 to 454 grains/1000 mm^2^ between 2012 and 2021. During the same period, common ragweed pollen levels rose from nearly undetectable to 8–22 grains/1000 mm^2^ by 2021, with further doubling predicted for 2024–2025 [23]. Data from the joint monitoring network of the Beijing Meteorological Service Center and the Department of Allergy, Beijing Tongren Hospital, Capital Medical University, demonstrated that pollen concentrations of both species increased in 2025 relative to 2024: common ragweed (540–720 vs. 410–560 grains/1000 mm^2^) and mugwort (2320–2650 vs. 2050–2380 grains/1000 mm^2^). These rising pollen loads constitute the fundamental driver of the 2025 rebound in sensitization‐positive rates to common ragweed and mugwort pollen allergens. The observed rebound in sensitization rates to these two allergens in 2025 underscores the need for targeted weed control strategies to effectively lower the incidence of allergies induced by common ragweed and mugwort pollen allergens.

The study found that positive rates varied across different age groups. The positive rates for common ragweed and mugwort pollen allergens showed a gradual decrease with increasing age, with the peak occurring in the 6–15 age group. Although SPT results in northern China indicate that weed pollen allergen is the primary cause of sensitization in the 4‐year‐old population [18], there are discrepancies in the peak age of sensitization and age‐related trends among different research findings. For instance, a study conducted in Beijing revealed that the 10–19 age group had the highest positive rate to inhalant allergens [17], and a study from the United States showed that sensitization rates peaked in the 10–19 age group and declined with increasing age [24]. This is inconsistent with the peak sensitization age for common ragweed and mugwort pollen allergens observed in this study. A retrospective analysis of SPT results from allergic patients at Beijing Shijitan Hospital, covering the period from January 2017 to December 2019, showed that the peak positive rate for common ragweed pollen allergens occurred in the 30–39 age group [18], and the highest prevalence of AR in Shanghai, China, was observed among patients aged 7–12 years [25]. Additionally, a 12‐year single‐center study found that the positive rate to common ragweed and mugwort pollen allergens peaked in the 20–29 age group and then gradually decreased with age, while the positive rates to the allergens increased gradually [26]. A study from Pakistan involving 2796 children under 18 years of age found that sensitization to environmental allergens was higher in younger children [27]. Both our findings and most of the previous literature demonstrated that sensitization rates decline with increasing age, which may be attributed to immunosenescence or the development of immune tolerance due to cumulative exposure in older adults. The discrepancies in peak age groups and age‐related sensitization trends reported across different studies are likely underpinned by continuous alterations in local vegetation composition and ambient environmental factors. Specifically, environmental perturbations can elicit de novo allergic sensitization to common ragweed and mugwort pollen allergens among elderly and pediatric populations with no prior sensitization history, thereby contributing to the progressive evolution of population‐level sensitization features for these two key aeroallergen sources. This finding highlights the necessity of conducting regional and dynamic studies to clarify the sensitization features of these allergens and their distribution across local populations.

In this study, male participants exhibited significantly higher positive rates for common ragweed and mugwort pollen allergens relative to female participants. Skin prick testing of allergic patients at Beijing Shijitan Hospital spanning from January 2017 to December 2019 demonstrated a significantly elevated positive sensitization rate to common ragweed pollen allergens in females relative to males [18]—a result conflicting with our study’s observations. In contrast, another Beijing‐based study found males to be more susceptible to mugwort pollen allergen sensitization than females [17], with a 12‐year single‐center study further reporting markedly higher positive rates to both common ragweed and mugwort pollen allergens in males [26]. A study from Pakistan involving 2796 children under 18 years of age found that male children had higher overall allergen sensitization rates [27]. A study from the United States revealed that males had significantly higher positivity rates for tree pollen allergens than females. These latter studies are consistent with the conclusions of the present study. Additionally, research shows that males tend to have higher IgE values than females, and this is true even if they don’t have allergies [28], and this trend was also observed in children in central China. The boys had significantly higher tIgE levels than the girls [29]. Collectively, these findings confirm that males have higher sensitization to allergens than females, and sensitization to common ragweed and mugwort pollen allergens is also higher in males than that in females. This difference between genders needs to be studied at a deeper level.

In this study, it was also found that the sensitization characteristics of common ragweed and mugwort pollen allergens were highly consistent: the peak periods of sensitization were the same; the annual trends in positive sensitization rates were consistent, with all rates decreasing progressively each year and then showing a rebound in 2025; the positive rate in males was higher than that in females; the peak age of sensitization was 6–15 years; and 55.83% of all positive patients were sensitized to both common ragweed and mugwort pollen allergens simultaneously. According to research findings, among children diagnosed with severe pollen allergies, 76.9% were cosensitized to common ragweed and mugwort pollen allergens [30]. Both common ragweed and mugwort are Asteraceae species. Numerous studies have confirmed an increased likelihood of cross‐reactivity among pollen allergens from plants of the same family, as evidenced by several key observations: a Dongying‐based study in China revealed a significant positive correlation between sensitization to birch and poplar pollen allergens [31]; honey ingestion can trigger cross‐reactive allergic manifestations in individuals sensitized to common ragweed and mugwort pollen allergens [32]; and extensive cosensitization patterns have been observed across weed pollen allergens [33]. Panallergens present in common ragweed and mugwort pollen allergens are responsible for cross‐reactivity and pollen‐food allergy syndrome [34]. Thus, molecular allergy diagnosis can mitigate misdiagnosis and enable the precise identification of primary allergen sources for immunotherapy intervention. Yet, even state‐of‐the‐art molecular allergy diagnostic techniques cannot entirely eliminate cross‐reactivity; a typical example is the Art v 1 component in mugwort pollen, which can elicit allergic reactions to common ragweed pollen allergens [35]. Accurate identification and detection of pollen allergenic components can not only enable the effective exclusion of cross‐reactivity but also provide a scientific basis for predicting allergic symptom phenotypes: patients testing negative for polcalcins predominantly exhibited cutaneous symptoms, while polcalcin‐positive patients tended to manifest more respiratory symptoms [36]. Molecular diagnosis, particularly component‐resolved diagnosis using specific mugwort pollen allergens (e.g., Art d 1, Art d 2, Art d 3, Art d 7, and Art d 9), not only significantly enhances diagnostic accuracy but also aids in predicting disease severity [37]. An Italian study has revealed that sensitization to both Art v 1 (mugwort) and Amb a 4 (common ragweed) pinpoints a distinct subgroup with a significantly elevated risk of food reactions. The findings indicate a strong association, with an odds ratio of 6.9 (*p* < 0.0001), highlighting the clinical importance of this dual‐sensitization profile [34]. Nevertheless, the achievement of highly refined detection of pollen allergen components remains a major challenge that requires further research and development.

Sensitization to pollen allergens is characterized by marked regional variability. In this study, we delineated the sensitization features of common ragweed and mugwort pollen allergens in northern Beijing during recent years through a comprehensive retrospective analysis of their sensitization profiles, with key findings as follows: since 2020, the overall positive sensitization rates to these two allergens have displayed a progressive downward trend before rebounding in 2025; male subjects had a significantly elevated positive sensitization rate relative to females; mugwort pollen allergens showed a higher positive rate than common ragweed pollen allergens; mugwort pollen allergens exerted a more potent stimulatory effect on the body’s production of sIgE; and there was a high prevalence of cosensitization to common ragweed and mugwort pollen allergens, an observation that is consistent with the reports in the literature. Collectively, these findings provide valuable evidence and a critical reference basis for the targeted prevention and personalized management of pollen allergen‐induced allergic diseases in the Beijing region.

Due to limitations of the dataset, we excluded previously diagnosed allergic patients, subjects with mild symptoms or asymptomatic sensitization, and those who declined sIgE tests, which led to an underestimation of the real population sensitization rate.

## Author Contributions

Zhipeng Zhao and Runqing Li conceptualized and wrote the manuscript. All other authors participated in the operation and data collection.

## Funding

This study was funded by the Incubation Program Project of Beijing Municipal Administration of Hospitals of China (Grant PG2018013), the Education Reform Project of Tsinghua University (Grant ZY01_02), and the Beijing High‐level Public Health Technical Personnel Project (Grant 2023‐03‐03).

## Disclosure

All authors edited the manuscript and approved the final version.

## Ethics Statement

The study protocol was reviewed and approved by the Institutional Review Board of Beijing Tsinghua Changgung Hospital. The ethical approval number is 24761‐4‐01.

## Consent

The authors have nothing to report.

## Conflicts of Interest

The authors declare no conflicts of interest.

## Data Availability

The data that support the findings of this study are available upon request from the corresponding author. The data are not publicly available due to privacy or ethical restrictions.
